# Autonomic Dysfunction in a Patient Initially Diagnosed With Parkinson’s Disease Who Subsequently Developed Systemic Amyloidosis

**DOI:** 10.7759/cureus.87116

**Published:** 2025-07-01

**Authors:** Joshua S Helali, Anuj Mahindra, Rajeev Mohan, Nelson Hwynn

**Affiliations:** 1 Department of Internal Medicine, Scripps Clinic, San Diego, USA; 2 Department of Hematology and Oncology, Scripps Clinic, San Diego, USA; 3 Department of Cardiology, Scripps Clinic, San Diego, USA; 4 Department of Neurology, Scripps Clinic, San Diego, USA

**Keywords:** amyloidosis, case report, dysautonomia, neurodegenerative disorders, parkinson's disease

## Abstract

Parkinson’s disease and closely related Parkinsonian neurodegenerative diseases are frequently associated with neurogenic orthostatic hypotension. In many cases, the dysautonomia would be attributed to Parkinson’s disease or its closely related neurodegenerative mimics. We present our experience diagnosing and managing a patient with dysautonomia attributed to an initial diagnosis of Parkinson’s disease and systemic amyloidosis, which the coexistence of these two diseases have not been previously reported in the literature.

Our 73-year-old patient developed levodopa-responsive symptoms initially suggestive of Parkinson’s disease. Within three years, he developed progressively worsening neurogenic orthostatic hypotension and, eventually, symptomatic bradycardia requiring a pacemaker, and was found to have a condition causing the dysautonomia other than Parkinson’s. Further workup led to a concurrent diagnosis of amyloid light-chain amyloidosis, which was successfully treated with chemotherapy. Despite being in remission of the amyloidosis and having satisfactory control of the Parkinson’s motor symptoms, his neurogenic orthostatic hypotension continued to be severely disabling, and overall functioning continued to decline.

Although neurogenic orthostatic hypotension is common in Parkinson’s disease and closely related neurogenerative disorders, there may be clinical features discordant with a neurodegenerative disorder that could lead to an alternative explanation for the dysautonomia. This patient not only fell into the usual Parkinsonism with dysautonomia differential diagnosis but also suggests that Parkinsonian conditions and systemic amyloidosis may exert a synergistic effect on morbidity, explaining why management of dysautonomia in these patients and reducing disability may be a significant challenge.

## Introduction

Orthostatic hypotension is prevalent in elderly populations and increases fall risk, hospitalizations, and mortality [1,2]. While nonneurogenic etiologies of orthostatic hypotension, such as dehydration, anemia, or medication side effects, may resolve after addressing the inciting cause, neurogenic orthostatic hypotension is a manifestation of autonomic dysfunction. A multitude of conditions can result in neurogenic orthostatic hypotension, including diabetes mellitus, neurodegenerative disorders such as Parkinson’s disease, dementia with Lewy bodies, multiple systems atrophy, small fiber neuropathy, systemic/cardiac amyloidosis, or autoimmune/paraneoplastic conditions [3].

Determining the etiology of dysautonomia can have implications for pharmacologic therapy and managing patient expectations, and can prompt diagnostics and treatment of other contributing conditions [4,5]. Peripheral autonomic neuropathies such as those caused by Parkinson’s disease and Lewy body dementia may respond to norepinephrine precursors such as droxidopa, whereas central dysautonomias such as multiple system atrophy (MSA) may have better response to norepinephrine reuptake inhibitors [4,5].

Workup of neurogenic orthostatic hypotension requires consideration of neurological and nonneurogenic processes known to cause autonomic dysfunction. We, therefore, rely on experiences of how different etiologies of dysautonomia progress and respond to different therapies. We present our experience diagnosing and managing a patient with dysautonomia attributed to both amyloidosis and Parkinson’s disease, a unique co-occurrence that has not been described in the medical literature.

## Case presentation

Our patient was initially diagnosed with Parkinson’s disease at age 73 years old with initial symptoms of micrographia and asymmetric resting tremor of the left hand, shuffling gait (but no falls), and findings of cogwheel rigidity. He had normal eye movements, normal motor strength, deep tendon reflexes, and coordination on examination, without obvious loss of sensation to cold temperature or vibration in his legs in a neuropathy fashion. He was started on carbidopa/levodopa and experienced significant symptomatic improvement of tremor and gait until three years after diagnosis, when he began to experience worsening gait instability. Rasagiline was added to his medication regimen at that time with symptomatic improvement for an additional year, until he began to report new onset severely symptomatic orthostatic lightheadedness (supine blood pressure 158/76 mmHg with pulse 59 bpm, immediately upon standing blood pressure 116/69 with pulse 63, and three minutes after standing blood pressure 85/47 mmHg with pulse 55 bpm). During this time, he was found to be inappropriately bradycardic as well (heart rate 30s bpm with sinus pauses of 3.6 seconds), attributed to sick sinus syndrome. Echocardiogram showed moderate concentric hypertrophy. He had no known history of cardiac disease before this. He subsequently had a pacemaker implanted, and his complaints of orthostatic dizziness again temporarily improved.

**Figure 1:**
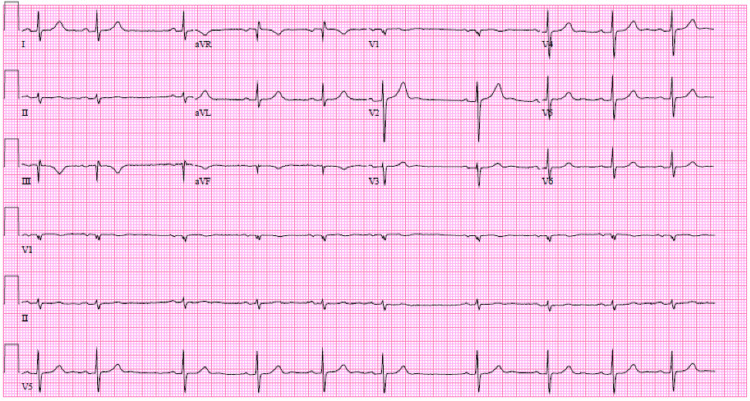
Normal sinus rhythm with sinus arrhythmia, which notably does not meet ECG criteria for chamber enlargement nor criteria for low-voltage aVR: augmented vector right; aVL: augmented vector left; aVF: augmented vector foot

**Figure 2:**
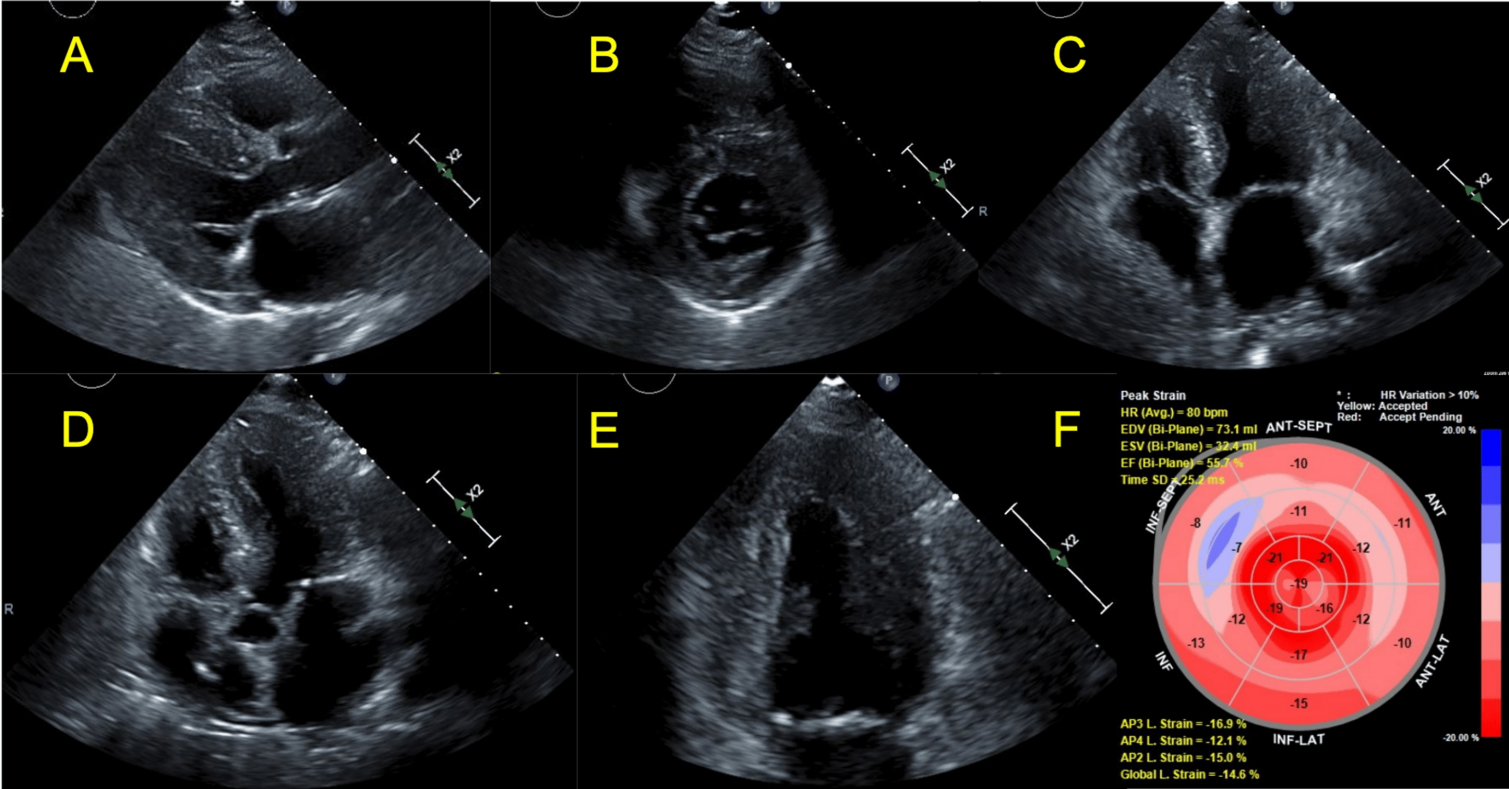
(A) Parasternal long-axis view showing thickened left ventricular septum and inferior wall. (B) Parasternal short-axis view at the level of the mitral valve showing concentric thickening of the left ventricle. (C) Apical four-chamber view showing thickening of the inferoseptal and lateral walls. (D) Apical five-chamber view showing thickening of the anteroseptal and inferolateral walls. (E) Apical two-chamber view showing thickening of the anterior and inferior walls. (F) Global longitudinal strain is reduced at -14.6% (normal is negative values greater than 18%). Strain values are relatively more normal at the apex (“apical sparing”). These findings are typical of cardiac amyloidosis

**Figure 3:**
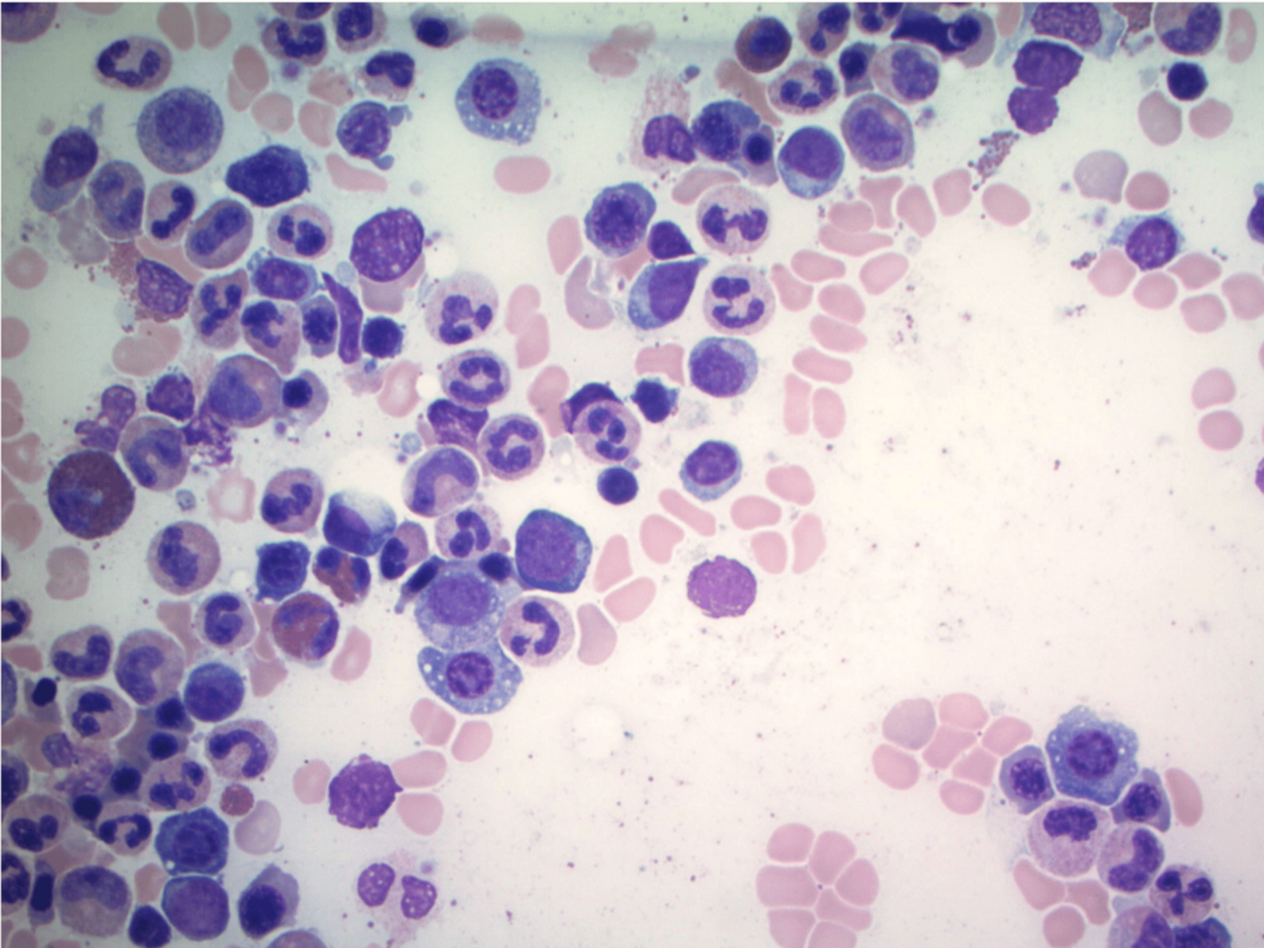
Bone marrow aspirate smear with scattered atypical plasma cells, with the loss of eccentricity and cytoplasmic vacuoles

**Figure 4:**
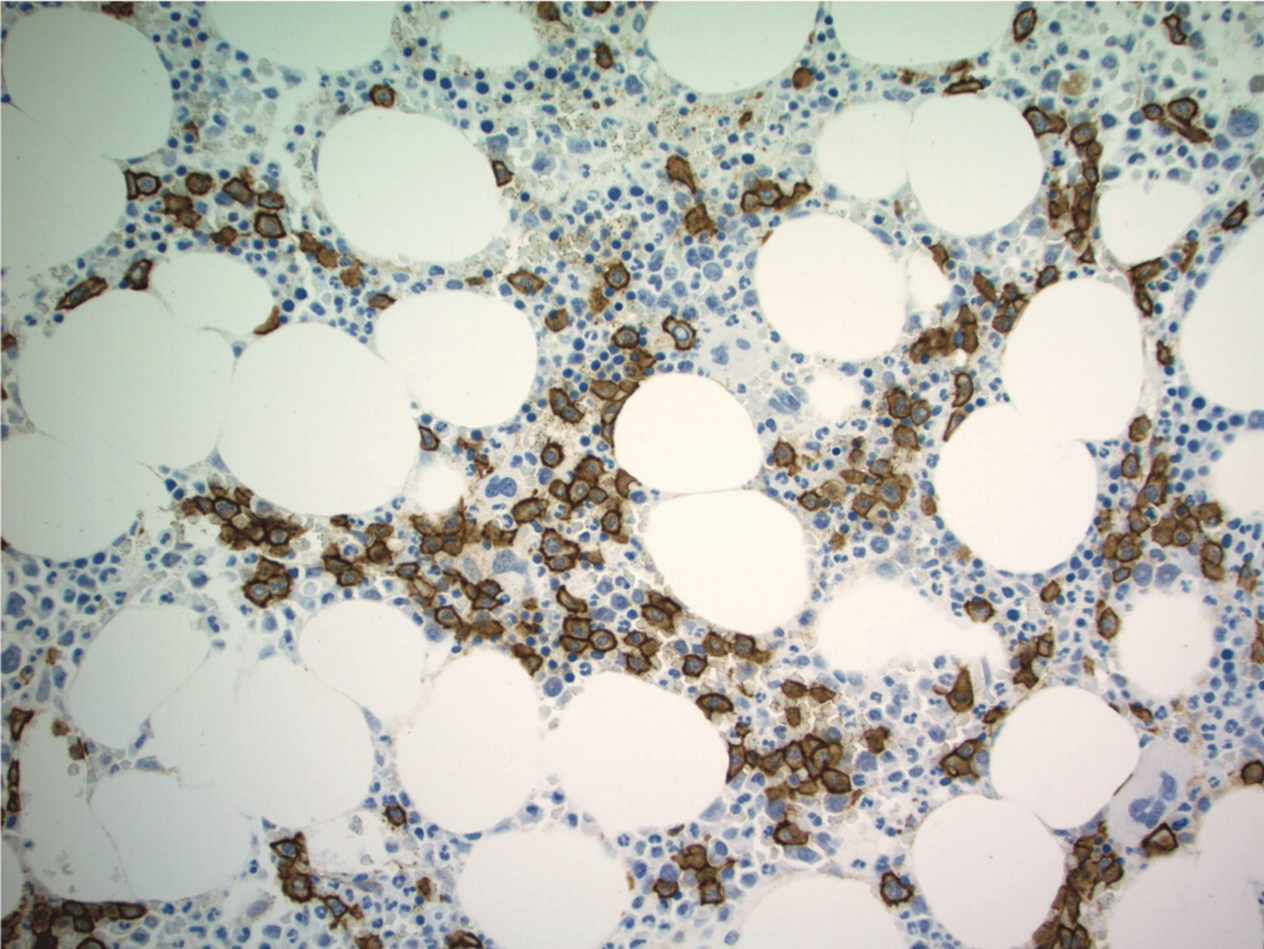
CD138 immunostain performed on a biopsy core that highlights increased interstitial plasma cells

**Figure 5:**
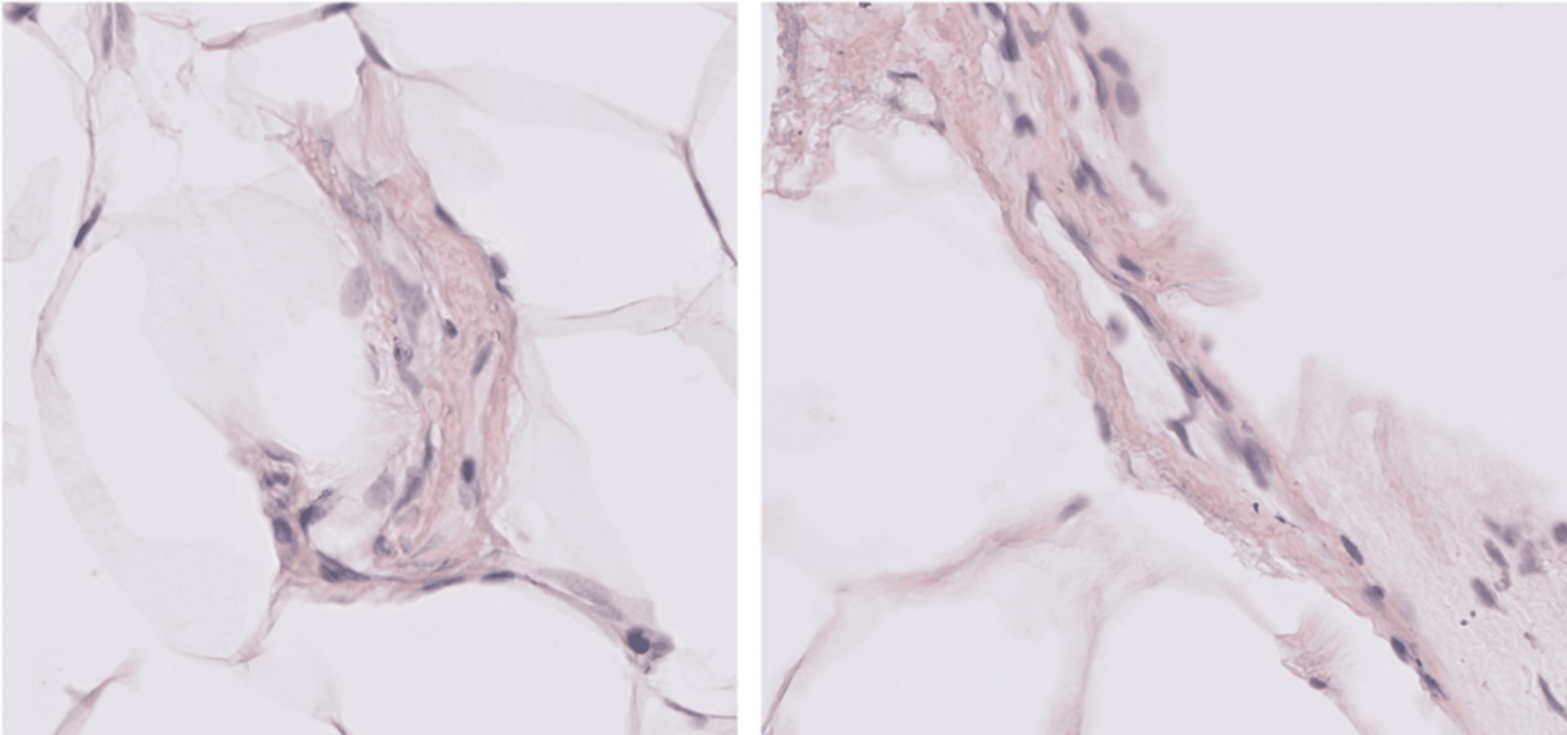
Abdominal fat pad biopsy slides (left and right) both showing orangeophilic perivascular deposition of amyloid, focally identified with Congo red stain

A few months later (almost five years after diagnosis), his orthostatic symptoms worsened again. At this point, he was actively staying well-hydrated, and his pacemaker was functioning properly. He had insulin resistance with hemoglobin A1C of 6.1% at the time (no overt diabetes). His workup showed unremarkable serum and urine protein electrophoresis, but with markedly elevated serum lambda free light chains. The 12-lead EKG showed unremarkable findings (Figure 1). Serial echocardiograms had shown progression from moderate to severe concentric hypertrophy with apical sparing on longitudinal strain, and ejection fraction reduction from 62% to 49% (Figure 2). There was noted concern for an infiltrative myocardial process, specifically cardiac amyloidosis, given that an apical sparing pattern on echocardiography is particularly sensitive (93%) and specific (82%) for cardiac amyloidosis [6,7]. He then underwent bone marrow biopsy, which revealed 15% plasma cells with aspirate smear with scattered atypical plasma cells with loss of eccentricity and cytoplasmic vacuoles and immunostaining showing increased interstitial plasma cells (Figures 3, 4), and subsequent fat pad biopsy confirmed a diagnosis of amyloid light-chain (AL) amyloidosis (Figure 5).

He was treated with six cycles of bortezomib, dexamethasone, and cyclophosphamide. By cycle 3, his serum light chains normalized. Bone marrow biopsy after cycle 6 showed that he was in successful remission, and he has subsequently remained in remission. Despite this, the patient’s symptomatic orthostatic hypotension continued to be debilitating. He was hospitalized for another fall attributed to orthostasis shortly after AL amyloidosis remission. He was also hospitalized twice in the subsequent six months for heart failure exacerbations in the setting of diastolic heart failure. He had regular follow-up appointments with his neurologist and heart failure specialist, including implantation of a CardioMems (Abbott, Atlanta, GA) device for aid in volume status optimization. Based on subsequent ambulatory pulmonary artery pressure monitoring from his CardioMems device, his heart failure team judged him to be generally euvolemic, and a strategy of fluid liberalization to 2-2.5 L/day with as-needed hydralazine for systolic blood pressure above 180 mmHg and diastolic blood pressure above 100 mmHg was adopted. He had tried and failed droxidopa and midodrine, with doses of these blood pressure-elevating medications significantly limited by the concern of exacerbating his amyloidosis heart failure via increases in his blood pressure. He was also unable to start fludrocortisone for this same reason. He had also been managed with supportive measures, including compression stockings, physical and occupational therapy. Unfortunately, despite the best medical treatments, by year 8 after developing Parkinson's symptoms, his symptoms had progressed to the point of lightheadedness with sitting and standing, and he was becoming severely deconditioned, which contributed to his disability. Intrapulmonary pressures measured by CardioMems continued to suggest optimal volume status. His Parkinsonian motor symptoms (tremor, bradykinesia, cogwheel rigidity) were otherwise felt to be adequately controlled on dopaminergic treatment.

## Discussion

This report provides an eight-year longitudinal account of this patient’s clinical course and our evolving diagnostic rationale. Every patient has their own circumstances and comorbidities, which limit any case report’s generalizability.

While autonomic dysfunction is common in Parkinson’s disease, other notable Parkinsonisms are often thought of, such as multiple systems atrophy or Lewy body dementia. Systemic amyloidosis concomitant with Parkinson’s has not been reported in the literature. Our patient underwent extensive measures to address his cardiac issues contributing to orthostasis, including pacemaker placement and a CardioMems device to ensure appropriate cardiac filling pressures. As his orthostatic symptoms continued to worsen despite optimization of nonneurogenic contributors, it would eventually become clear that he had another process going on other than Parkinson’s disease.

Parkinson’s disease is known to cause autonomic dysfunction that could progress despite acceptable control of standard motor features of Parkinson’s symptoms [4,8,9], as was the case in our patient. Our first clue toward another contributing disease process was his need for a pacemaker. Patients with neurodegenerative disorders such as Parkinson’s disease, Lewy body dementia, and MSA generally do not receive pacemakers for dysautonomia alone [10,11]. Dysautonomia from amyloidosis can potentially be overlooked in patients with Parkinson’s disease, and we relied on other systemic features to help make the diagnosis in this case.

The timing of the onset of our patient’s rapidly progressive neurogenic orthostatic hypotension, just one year before AL amyloidosis diagnosis, suggests amyloidosis may be a significant contributor to his dysautonomia at that time. However, we cannot rule out the possibility of an underlying Parkinson-plus syndrome, such as multiple systems atrophy or Lewy body dementia, evolving at the same time as his other features. However, despite his AL amyloid being in remission, his disability from orthostatic dizziness continued. There is a published case report of another patient with AL amyloidosis having significant improvement with midodrine and droxidopa, but our patient, unfortunately, did not have the same therapeutic outcome with these medications [12]. This may be due to his neurodegenerative disorder, superimposed on damage already caused by AL amyloid deposition around peripheral nerves. Despite amyloid disease remission after treatment, nerve damage from amyloidosis is not expected to improve to a significant degree [13]. Additionally, part of the pathophysiology of neurogenic orthostatic hypotension in Parkinson's is the accumulation of alpha-synuclein in peripheral nerves [4,9,14,15]. There is no literature about a possible synergistic effect of Parkinson's and AL amyloid on the development of dysautonomia. This may be a direction for future research.

## Conclusions

Patients with Parkinsonian conditions who have neurogenic orthostasis may have Parkinson’s disease or other neurodegenerative parkinsonisms considered as the cause of the dysautonomia. However, clinical features discordant from typical neurodegenerative disorders, such as the need for a pacemaker, should prompt additional work-up. It is likely that having both conditions concurrently exerts a pathophysiologic synergistic effect on the autonomic nervous system. In our patient, commonly used medications such as droxidopa and midodrine, unfortunately, did not provide satisfactory symptomatic improvement, and his condition progressed despite successful treatment of AL amyloidosis into remission. The treatment of the patient’s neurogenic orthostatic hypotension was complicated by amyloidosis, causing cardiac failure.
